# Notes on Mucous Disease

**Published:** 1872-05

**Authors:** Walter Whitehead

**Affiliations:** Edinburgh


### NOTES ON MUCOUS DISEASE.

BY WALTER WHITEHEAD, F. R. C. S., EDINBURGH.

This disease "is characterized by the secretion of mucus of an abnormal character over mucous surfaces, in which condition the mucus is prone to consolidate into masses, shreds, or tubular casts. These concretions form and exfoliate periodically, each exfoliation being critical, and immediately followed by an amelioration of the symptoms which aggravated up to this point. This critical period is accompanied by pains of a spasmodic character, and of variable intensity."

The mucous may be discharged from the bowel—1st, in a more or less inspisated condition; 2d, in a concrete or semi-solid condition. The mucous concretions are not always easily recognized when mixed with the motions, but their nature may generally be recognized if they are floated in water. In some cases they are found in masses the size of walnuts; in others we get extensive membranes, thick, and of considerable firmness and tenacity. The formation, exfoliation, and expulsion of these mucous structures observe a regular order; each stage of the disease being attended by characteristic symptoms, which have been described in the notice alluded to. A microscopic examination of the concretions reveals the following construction: "They are composed of layers of a semi-solid, transparent, hyaline, amorphous matrix, in which spherical cells are imbedded together with epithelial cells in various stages of growth, free nuclei, crystals of triple phosphate, and undigested and undigestible matter."

A chemical examination of the mucous casts, made by Andrew Clark, shows that the matrix "is fibrillated by acetic acid. Careful washing and compression yielded a fluid abundantly coagulable by heat and nitric acid. Prolonged digestion of the casts at an elevated temperature in solution of nitre produced no fluid abundantly coagulable, or precipitable by acetic acid." The disease occurs much more frequently in women than in men, and generally in persons between thirty and forty years of age. The phlegmatic temperament seems to predispose to it. Among the more common symptoms are dyspepsia, palpitation of the heart, a depresssd and desponding condition of mind, a feeling as if the digestive tract were raw in places, and a variable condition of the tongue. In addition to these, successive crops of eruptions, and the ulcerations left by them, can frequently be detected in the inside of the lips, cheeks, gums, and tongue. Occasionally, too, membranes resembling those discharged from the bowel are expelled from the bladder, and in women, during menstruation, from the uterus.

Mr. Whitehead sums up his conclusions respecting the disease as follows:

"1. That the mucous membranes, like the skin (and is not the one looked upon as inversion of the other?), is prone, under certain conditions, in certain constitutions, to develop products unnatural to their functions. It is not natural for the skin to produce eczema, neither is it natural for mucous surfaces to produce mucus in a concrete form.

"2. (A.) That the proximate cause of the symptoms referable to this disease is the hypersecretion and accumulation of mucus on the free surface of mucous membranes; such accumulations sheathe and prevent the healthy performance of the functions natural to the part, and thus induce immediate and remote results, the effect of such suppressed functions. (B.) That this hypersection indicates a want of balance between nerve force and germinal matter. (C.) That the nerve force is perverted by irritation. (D.) That the exciting causes are numerous. (E.) That it is a character of mucous secretions under the influence of irritation for its cell elements to increase, and its viscidity to diminish. (F.) And that in the disease in question the prolific cell formations become entangled in the albuminous fluid in which they are found, and present the membranous structures before referred to."

The prognosis is not unfavorable in recent cases, or those in which the cause of the irritation can be removed, but whenever the disease becomes chronic, it is generally found intractable to remedies. The principal points in the treatment are as follows:

1. "Discover and counteract any cause, either in direct contact, or in the immediate vicinity of the secreting surface, which can be traced as a source of irritation, such as accumulations of scybala, an inflamed pile, or the use of any drugs known to be hurtful in this condition of the system." This applies with especial force to drastic purges. Belladonna and enemata may be given to relieve constipation. 2. "Reinvigorate the strength and allay the nervous irritability. 3. Relieve the accumulated mucus; and 4. Prevent its re-formation." To fulfil the second indication, bromide of potassium, the various preparations of iron, attention to the skin, and the regulation of the diet are recommended. To meet the third, injections of solutions of soda, potassa, and lime may be used, and the re-formation of mucus will be best prevented by the use of astringent solutions. The paper is illustrated by ten cases, and appended to it is a full biography, which adds very materially to its value.—*Manchester Medical Reports, in Half-Yearly Abstract.*

